# Multifunctional metasurface lens for imaging and Fourier transform

**DOI:** 10.1038/srep27628

**Published:** 2016-06-08

**Authors:** Dandan Wen, Fuyong Yue, Marcus Ardron, Xianzhong Chen

**Affiliations:** 1 SUPA, Institute of Photonics and Quantum Sciences, School of Engineering and Physical Sciences, Heriot-Watt University, Edinburgh, EH14 4AS, UK; 2 Renishaw PLC, Research Avenue North, Riccarton, Edinburgh EH14 4AP, UK

## Abstract

A metasurface can manipulate light in a desirable manner by imparting local and space-variant abrupt phase change. Benefiting from such an unprecedented capability, the conventional concept of what constitutes an optical lens continues to evolve. Ultrathin optical metasurface lenses have been demonstrated based on various nanoantennas such as V-shape structures, nanorods and nanoslits. A single device that can integrate two different types of lenses and polarities is desirable for system integration and device miniaturization. We experimentally demonstrate such an ultrathin metasurface lens that can function either as a spherical lens or a cylindrical lens, depending on the helicity of the incident light. Helicity-controllable focal line and focal point in the real focal plane, as well as imaging and 1D/2D Fourier transforms, are observed on the same lens. Our work provides a unique tool for polarization imaging, image processing and particle trapping.

The reshaping of the light wavefront in traditional optical elements relies on gradual phase change, which is realized by light propagation through media of given refractive indices that can be engineered to control the optical path of light beams. Therefore, the deterministic functionality of an element is usually dictated by the surface topography. For example, a cylindrical lens can perform one-dimensional (1D) optical transformation while a spherical lens can perform two-dimensional (2D) transformation. Driven by device miniaturization and system integration, there is a huge interest in obtaining a lens with multiple functionalities, which can dramatically increase the functionality density of the optical system. The multi-focal lens^1^,^2^ and multi-order focusators^3^ represent the significant exploration in this field. However, these elements have a thickness much larger than light wavelength and suffer from narrow bandwidth since they are designed based on diffractive optics.

Metasurface, as a new type of artificially structured surface, has provided a new design methodology for ultrathin optical devices, allowing manipulation of light propagation to an unprecedented level by imparting local and space-variant abrupt phase change^4^,^5^,^6^,^7^,^8^,^9^,^10^,^11^,^12^,^13^,^14^,^15^,^16^,^17^,^18^,^19^. With the rapid development in metasurfaces, the conventional concept of what constitutes an optical lens continues to evolve. Ultrathin metasurface lenses have been demonstrated based on various nanoantennas such as V-shape structures^20^, nanorods^21^,^22^ and so on^23^,^24^,^25^,^26^. Dual-polarity flat lenses have been developed based on helicity-dependent phase discontinuities for circularly polarized light^27^. Specifically, the positive and negative polarity are interchangeable by controlling the helicity of the incident light. From the point of view of system integration and device miniaturization, a single device that can combine the functionalities of a spherical lens and a cylindrical lens is desirable. However, such a lens has not been developed yet.

Based on the unique design methodology presented in our recent work^28^, we experimentally demonstrate an ultrathin metadevice that integrates two different types of lenses and polarities in this paper. The geometric phase^17^,^29^,^30^,^31^,^32^,^33^,^34^,^35^ based metasurface lens can work either as a spherical lens or a cylindrical lens, depending on the polarization state of the incident light. A focal line and a focal point in the real focal plane are observed upon the illumination of right circular polarization (RCP) and left circular polarization (LCP), respectively. The unique properties of the lens in imaging and image processing for Fourier transform are explored. More importantly, the polarity of the multifunctional lens is changed when the helicity of the incident light is reversed, which shows polarization dependent imaging and Fourier transform properties. As a brand new device, the unique performance of such a multifunctional lens in imaging and image processing has potential application in particle trapping, imaging and information processing.

## Methods

**Figure 1:**
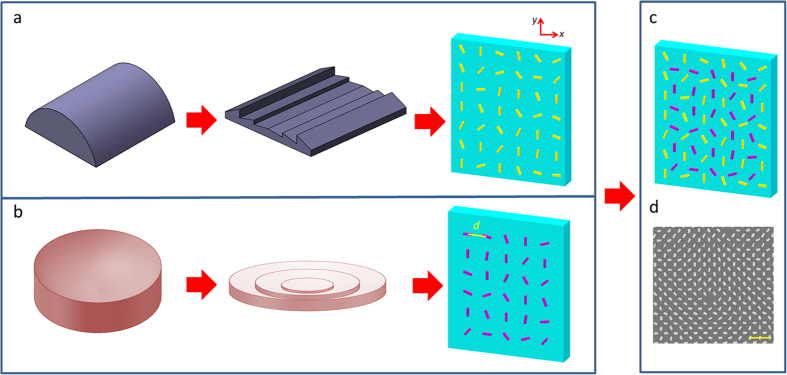
Different mechanisms for lens design. (**a,b**) The evolution process from a conventional lens and a Fresnel lens to an ultrathin metasurface lens. The lens types in (**a**,**b**) are cylindrical and circular, respectively. (**c**) The multifunctional lens is designed by integrating two metasurface lenses with different polarities onto a single metasurface. Two sets of nanorod arrays corresponding to a cylindrical lens and a spherical lens are merged together with a displacement vector of (*d*/2, *d*/2). *d* is the distance between neighboring antennas with a value of 500 nm along *x* and *y* directions. Each nanorod is 200 nm long, 80 nm wide and 40 nm high. The size of the multifunctional metasurface lens is 333 μm × 333 μm. (**d**) Scanning electron microscopy (SEM) image of part of the multifunctional lens. The scale bar is 1 μm in the SEM image.

In order to reduce the thickness of the traditional lens, Fresnel lens was invented by replacing a standard lens with a set of separate surfaces, with stepwise discontinuities between them. Metasurfaces can further reduce the thickness of the lenses due to their capability in abruptly changing the optical wavefront properties within a deep subwavelength range [Fig. 1(a,b)]. In this work, metasurfaces consisting of elongated gold nanorods are utilized^21^. When a nanorod is excited by the circularly polarized incident beam, the complex scattering coefficients for the incident components polarized along the two axes of the nanorod are different. Therefore the nanorod can partly change the incident light into its cross polarization states^36^ with an additional geometric phase of ±2*φ*, where *φ* represents the orientation of the nanorod and the sign ‘+’ or ‘−’ corresponds to the incident LCP or RCP light, respectively. By utilizing the geometric phase, it is very convenient to encode a sampled phase function onto an array of nanorods with the same geometry but varying orientations.

In order to create the multifunctional lens, two independent designs regarding the positive cylindrical lens and the negative spherical lens are firstly carried out. The phase function for the positive cylindrical lens is


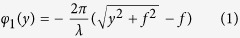


and that for the negative spherical lens is


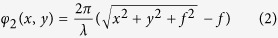


where *λ* is the incident wavelength and *f* is the focal length. Eqs (1) and (2) are sampled and encoded onto two separate nanorod arrays as shown in Fig. 1(a,b), respectively. Under the illumination of RCP light, the desired phase profile is generated by the scattered LCP light from the nanorod arrays. Based on the unique methodology we proposed recently^28^, the two nanorod arrays are then merged together to construct the multifunctional lens [Fig. 1(c)].

On the transmission side of the multifunctional lens, the divergent light from the negative spherical lens acts as a subtle background in comparison with the converging light from the positive cylindrical lens. On the contrary, the negative spherical lens “converges” and the positive cylindrical lens “diverges” the virtual light beams on the incident side. Due to the intrinsic properties of the geometric phase, the sign of the phase profile is flipped if the helicity of the incident circularly polarized light is reversed. The multifunctional lens turns into the combination of a negative cylindrical lens and a positive spherical lens if the incident light is changed from RCP to LCP.

## Results

### Focusing properties of the multifunctional lens

The scanning electron microscopy image of the fabricated metasurface is shown in Fig. 1(d). The gold nanorods are fabricated on the indium tin oxide coated glass substrate using the standard electron-beam lithography. In our experiment, the incident circularly polarized light is generated by a polarizer and a quarter-wave plate, and then impinges on the metasurface at normal incidence. The beam size is much larger than the metasurface to ensure near planar incident light. Another pair of polarizer and quarter waveplate are used on the transmission side to only collect the cross-polarized scattered light. An objective and a lens are used to perform the far field microscopy. The objective is mounted on a three-dimensional translation stage, which is used to adjust its position and capture the intensity distribution of the scattered light at different positions along the optical axis.

**Figure 2:**
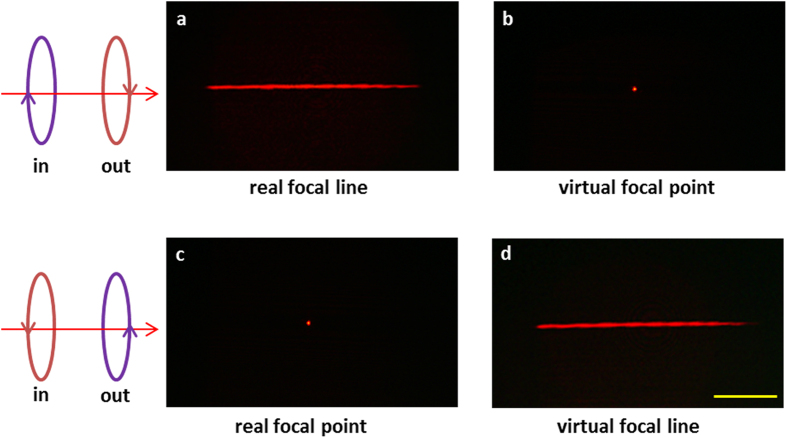
Experimental verification of the focusing properties of the multifunctional lens. (**a,b**) Under the illumination of RCP light at the wavelength of 640 nm, the scattered LCP light forms a real focal line on the transmission side at *z = f*, while it forms a virtual focal point on the incident side at *z *= −*f*. (**c,d**) If the incident/detected light are changed to LCP/RCP, a real focal point and a virtual focal line are observed at *z = f* and *z = *−*f*, respectively. The scale bar in the figure is 100 μm.

The spherical and cylindrical lens both have a focal length of *f* = 198 μm at the wavelength of 640 nm. When the incident/detected light is RCP/LCP, a clear focal line is observed at *z = f* on the transmission side [Fig. 2(a)]. By further shortening the distance between the objective and the metasurface, a virtual focal point is obtained at *z = −f* [Fig. 2(b)]. If the helicity of the incident/detected light are both reversed, a real focal point [Fig. 2(c)] and a virtual focal line [Fig. 2(d)] are obtained at *z = f* and −*f*, respectively. The experiment clearly verifies that the metasurface is the combination of a positive cylindrical lens and a negative spherical lens for RCP incident light. The polarities of both lenses are reversed when the helicity of the incident light is changed.

Upon the illumination of RCP light, it is transformed both by the positive cylindrical lens and the negative spherical lens simultaneously. Although the real focal line on the incident side is affected by the light emitting from the negative spherical lens, the intensity of the focal line is much higher than the background noise [Fig. 2(a)]. Similarly, although the light “emitting” from the positive cylindrical lens also contributes to the virtual focal point, a bright focal point with pure background can still be clearly observed [Fig. 2(b)]. Similar but opposite behaviour is observed for LCP incident light when the metasurface acts as a positive spherical lens and a negative cylindrical lens [Fig. 2(c,d)].

### Imaging properties of the multifunctional lens

**Figure 3:**
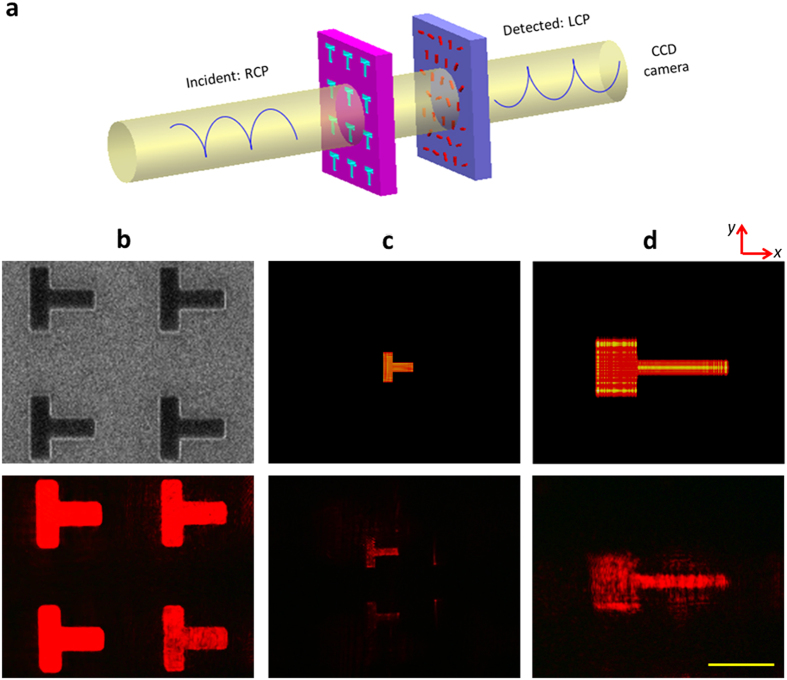
Imaging property of the multifunctional lens. (**a**) Schematic to show the experimental setup. (**b**) The SEM image of the T-shaped apertures (upper panel) and their CCD images without transformation by the multifunctional lens (bottom panel). Each T-aperture has the size of 50 μm × 50 μm. (**c,d**) Virtual images of the T-shaped apertures generated by (**c**) the negative spherical lens and (**d**) the positive cylindrical lens. The upper panel in each column shows the simulation results and the bottom panel shows the experimental results. The scale bar is 50 μm for all the images in (**b–d**).

As a lens, the imaging properties of the metasurface lens are our main concern. The object to be imaged is the T-shaped aperture array fabricated on a 40 nm thick chromium film. Each T-aperture has a size of 50 μm × 50 μm and the distance between the neighboring apertures is 100 μm along both directions in a square array. The scanning electron microscope image of the T-apertures is shown in the upper panel of Fig. 3(b). To verify the imaging properties of the multi-functional lens, the aperture array is placed at a distance away from the lens with an air gap between them [Fig. 3(a)]. The thickness of the air gap is firstly set to be about 105 um which is within the focal length of the lens, hence only virtual images of the T-apertures are observed. The incident RCP light firstly shines on the T-aperture array and part of the diffracted light is transformed by the lens. Due to the 2D transform of the spherical lens, a compressed virtual image is observed [Fig. 3(c)]. By further decreasing the distance between the objective lens and the multifunctional lens, a virtual image stretched along *x* direction is obtained due to the 1D transform of the cylindrical lens [Fig. 3(d)]. The upper row of Fig. 3(c,d) are the simulated results based on the diffraction theory, and the bottom row shows the CCD images obtained in the experiment. They show reasonable agreement with each other from the point view of image shape and magnification. As mentioned above, the polarities of the cylindrical lens and the spherical lens are reversed when the incident light is changed from RCP into LCP. A 1D compressed virtual image formed by the negative cylindrical lens and a 2D magnified virtual image formed by the positive spherical lens are observed upon the illumination of LCP incident light (see supplementary Fig. S1).

**Figure 4:**
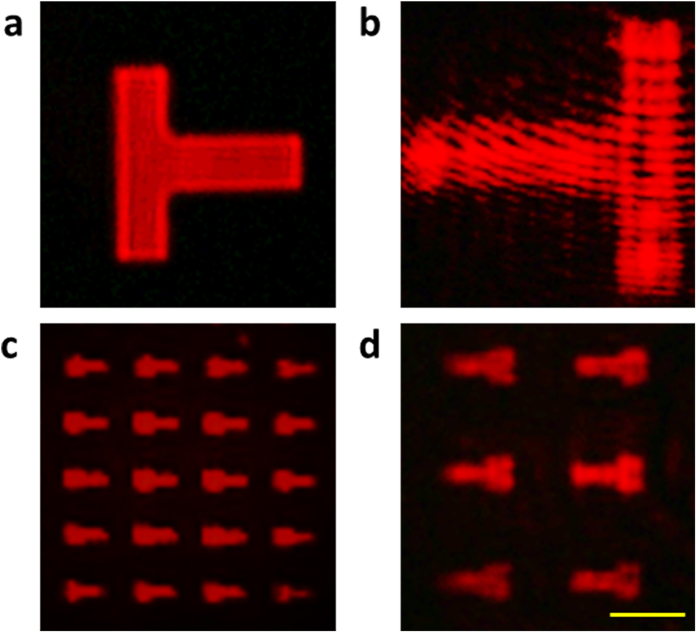
Real images obtained on the multifunctional lens under the LCP incident light. (**a**) CCD image of a large T-shaped aperture (50 μm × 50 μm) without transformation by the multifunctional lens. (**b**) The inverted real image of the T-shaped aperture in (**a**). (**c**) CCD image of the small T-shaped apertures (11 μm × 6 μm) without transformation by the multifunctional lens. (**d**) The inverted real images of the T-shaped apertures in (**c**). The scale bar is 20 μm for all the images. The magnifications of the two real images are both about 1.5 in (**b**,**d**) in comparison with their respective original objects.

Apart from the virtual images formed by the multifunctional lens, the magnified real images of the object are also obtained by increasing the thickness of the air gap to be larger than the focal length *f* but less than 2*f*. Since only the positive lens can form real images on the transmission side, the images formed by the positive spherical lens are shown in Fig. 4(b) when the incident light is LCP. Another T-aperture array with much smaller shapes (11 μm × 6 μm) is used to further verify the imaging capability of the multifunctional lens. The magnified real T-images with clear outlines are shown in Fig. 4(d).

### Fourier transform by the multifunctional lens

**Figure 5:**
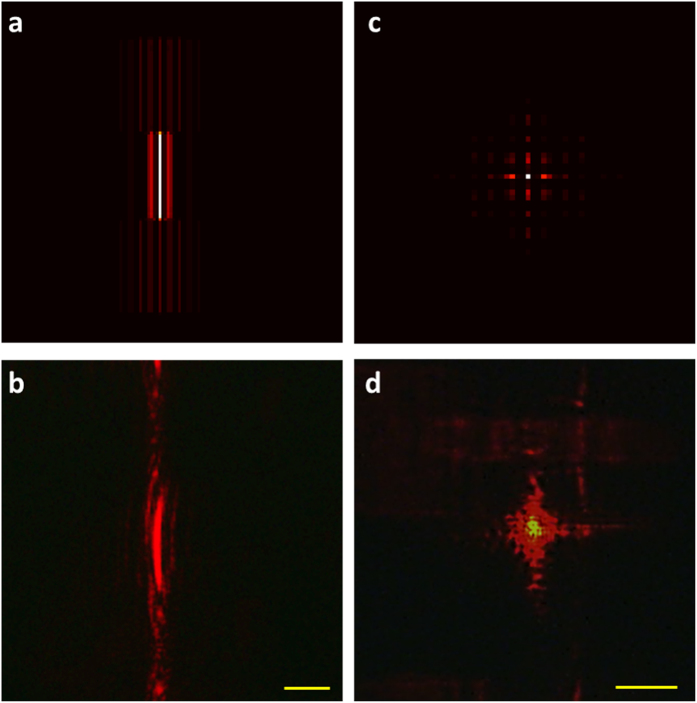
Fourier transform of the T-shaped aperture array by the multifunctional lens. The multifunctional metasurface lens functions as a positive cylindrical lens and a negative spherical lens upon the illumination of RCP light. (**a**) Simulated and (**b**) experimentally obtained 1D Fourier transform of the T-shaped aperture array. The CCD images are captured at the real focal plane. The Fourier transform is only performed along the horizontal direction. (**c**) Simulated and (**d**) experimentally obtained 2D Fourier transform of the T-shaped aperture array, which are captured at the virtual focal plane of the multifunctional lens. The scale bars in both (**b,d**) are 20 μm.

Apart from imaging, Fourier transform is another important functionality of the lens. The 1D and 2D optical Fourier transform can be carried out using the cylindrical lens^37^ and the spherical lens^38^, respectively. In the experiment, the thickness of the air gap is 105 μm (the thickness does not affect the Fourier transform property of the multifunctional lens). For the RCP incident light, the intensity distribution at the real and virtual focal planes represent the 1D and 2D Fourier transforms of the object by the positive cylindrical lens and the negative spherical lens, respectively. The 1D Fourier transform is only performed along the horizontal direction perpendicular to the focal line. Several lines are separated along the horizontal direction and they represent different spatial frequencies [Fig. 5(a,b)]. The cross-shaped pattern at the virtual focal plane shows the 2D Fourier transform of the T-shaped patterns [Fig. 5(c,d)] along two perpendicular directions. Figure 5(a,c) are the computational 1D and 2D Fourier transform of the T patterns using the fast Fourier transform. Figure 5(b,d) are the corresponding experimental results and show reasonable agreement with the simulation. In addition, the positions for 1D and 2D Fourier transforms are swapped upon the illumination of LCP light [see supplementary Fig. S2].

## Discussion

The uniqueness of the multifunction lens lies in the combination of a spherical lens and a cylindrical lens in a thin device. The unusual performance of such a brand new device has potential application in particle trapping, imaging and information processing. For example, the multifunctional optical tweezers can be realized with individually defined structures of each trap, which is switchable via the incident polarization state. Imaging systems that can be used to detect and analyze circularly polarized light have remarkable capabilities in biological applications^39^. Two spherical lenses with opposite polarities and different focusing property along the *x* axis^1^,^40^ (the optical axis is along *z* axis) can be integrated onto the same metasurface. If the incident light is linearly polarized and only the cross polarized scattered light is detected, the real images located at different positions corresponding to RCP and LCP light can be formed simultaneously.

The conversion efficiency is a very important parameter which affects the performance of the metasurface and it is defined as the ratio of the power of the helicity-changed light *I*_out_ to that of the input light *I*_in_. The helicity-unchanged light is filtered out by using a quarter waveplate and a polarizer. The conversion efficiency in the range of 640 nm ~1080 nm with a step size of 40 nm is measured and the peak efficiency is about 5% at 880 nm in these experiments. Although the geometric phase is independent of the incident wavelengths, the conversion efficiency of the metasurface is closely related to the incident wavelengths since the nanorods are intrinsically dispersive. The useful bandwidth of the metasurface is hence limited by the wavelengths where the efficiency is low.

Although the efficiency can be greatly improved using the reflective type metasurface^41^, the transmission type is more popular in real application. According to the general mechanism of improving the conversion efficiency^32^, the relationship *t*_uu_ + *t*_vv_* = t*_uv_ − *t*_vu_ = 0 should be satisfied for the transmission-type metasurfaces. Here, 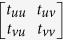 represents the Jones matrix of a nanoantenna and {u, v} are the reference coordinates. However, for the design in this work, we have *t*_uv_ − *t*_vu_ = 0 and *t*_uu_ + *t*_vv_ ≠ 0, which yields an unavoidable zero-order. To satisfy the above general relationship, a transmission-type dielectric metasurface^42^ might be a promising candidate to achieve higher efficiency.

The multifunctional metasurface lens in this paper is a combination of two different types of lenses with a real (virtual) focal point and a virtual (real) focal line located on different sides of the metasurface under the illumination of left (right) circularly polarized incident light. The combination design causes background noise, which decreases the signal-to-noise ratio of the image formed by the metasurface lens. However, the background noise can be dramatically deceased by utilizing the angle-multiplexing method^17^. For example, both the real focal point and real focal line are located on the transmission side, but they have different off-axis angles that are symmetric about the surface normal.

## Conclusion

In summary, we have experimentally demonstrated a multifunction metasurface lens consisting of nanorods with spatially variant orientation and operating at visible frequencies. The design is based on the abrupt phase change that occurs for the circularly polarized light. The observed focal line and focal point in the focal plane are interchangeable, depending on the helicity of the incident light. The unique properties of the metasurface lens in imaging and image processing in Fourier transform agree well with the theoretical prediction. This lens features the combination of two types of lenses operating with opposite incident helicities on the same metasurface, opening a new avenue to achieve a high density of functionality and effectively scaling down the size of photonic systems.

## Additional Information

**How to cite this article**: Wen, D. *et al.* Multifunctional metasurface lens for imaging and Fourier transform. *Sci. Rep.* **6**, 27628; doi: 10.1038/srep27628 (2016).

## Supplementary Material

Supplementary Information
