# Supplementary material for: Multifunctional metasurface lens for imaging and Fourier transform
Source: Sci Rep. 2016 Jun 8;6:27628. doi: 10.1038/srep27628 (PMC4897706; doi:10.1038/srep27628)
Supplement: Supplementary Information [file srep27628-s1.pdf]

Multifunctional metasurface lens for imaging and Fourier transform

Dandan Wen[1], Fuyong Yue[1], Marcus Ardron[2], Xianzhong Chen[1*]

1. *SUPA, Institute of Photonics and Quantum Sciences, School of Engineering and Physical Sciences, Heriot-Watt University, Edinburgh, EH14 4AS, UK*
2. *Renishaw PLC, Research Avenue North, Riccarton, Edinburgh EH14 4AP, UK*

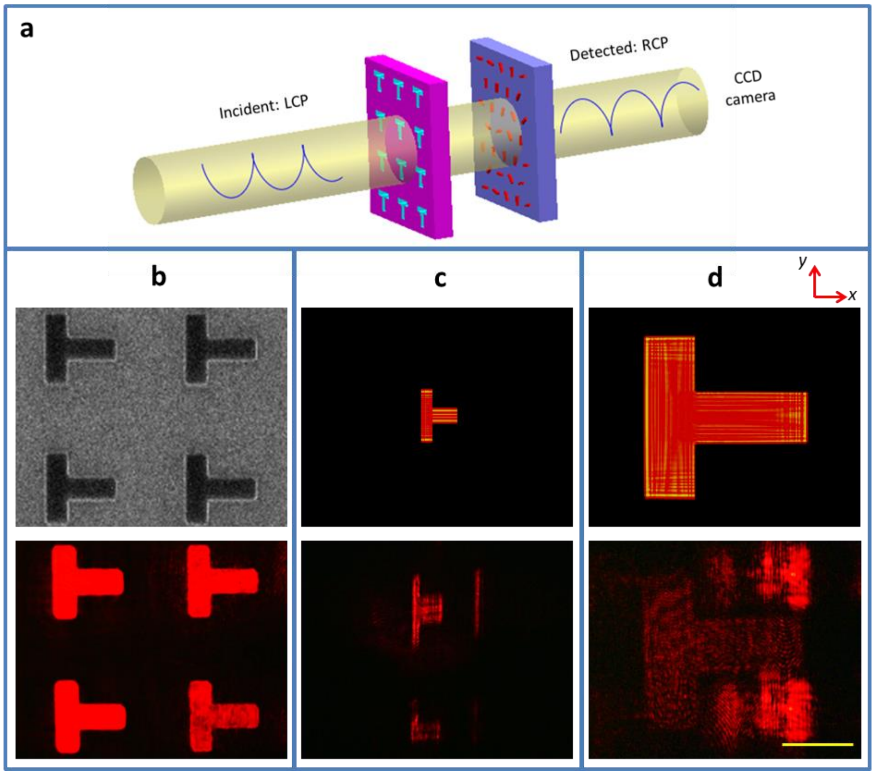

Figure S1 Imaging properties of the multifunctional metasurface lens. (a) Schematic to show the experimental setup. (b) The SEM image of the T-shaped apertures (upper panel) and their CCD images without transformation by the multifunctional lens (bottom panel). Each T-aperture has a size of 50μm×50μm. (c-d) Virtual images of the T-shaped

apertures generated by (c) the negative cylindrical lens and (d) the positive spherical lens. The scale bar is 50μm for all the images of (b), (c) and (d). The upper panel in each column shows the simulation results and the bottom panel shows the experimental results.

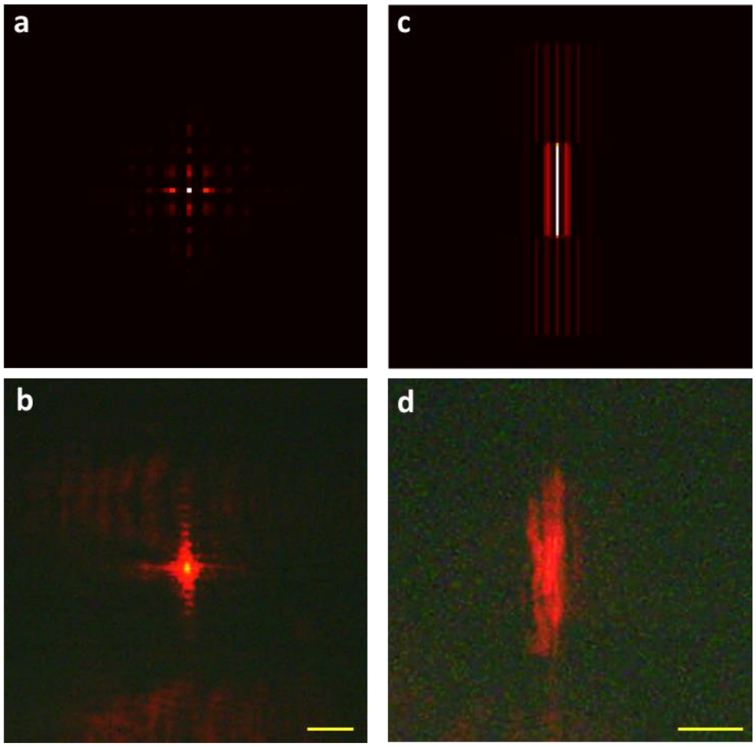

Figure S2 Fourier transform of the T-shaped aperture by the metasurface lens. The incident/detected light is LCP/RCP and the metasurface functions as a positive spherical lens and a negative cylindrical lens. (a) Simulated and (b) experimentally obtained 2D Fourier transform of the T-shaped patterns. The CCD images are captured at the real focal plane of the metasurface lens. (c) Simulated and (d) experimentally obtained 1D Fourier transform of the T-shaped patterns. The experimental results are captured at the virtual focal plane of the metasurface lens. The scale bars in (b) and (d) both represent 20 μm.
